# Nationwide outcomes of biliary atresia after Kasai portoenterostomy in Taiwan: the impact of postoperative cholangitis and corticosteroid use

**DOI:** 10.1007/s00383-026-06689-9

**Published:** 2026-10-20

**Authors:** Hsiao-Hui Yang, Wan Ting Huang, Jen-Hung Wang, Ming-Chun Chen, Shu-Hui Wen

**Affiliations:** 1 Division of Pediatric Surgery, Department of Surgery, Hualien Tzu Chi Hospital, Buddhist Tzu Chi Medical Foundation, Hualien, Taiwan; 2 School of Medicine, Tzu Chi University, Hualien, Taiwan; 3 Institute of Medical Sciences, Tzu Chi University, Hualien, Taiwan; 4 Epidemiology and Biostatistics Center, Hualien Tzu Chi Hospital, Buddhist Tzu Chi Medical Foundation, Hualien, 97004 Taiwan; 5 Department of Pediatrics, Hualien Tzu Chi Hospital, Buddhist Tzu Chi Medical Foundation, No. 707, Zhongyang Rd., Sec. 3, Hualien, 97004 Taiwan; 6 Department of Public Health, College of Medicine, Tzu-Chi University, No. 701, Zhongyang Rd., Sec. 3, Hualien, 97004 Taiwan

**Keywords:** Kasai portoenterostomy, Cholangitis, Liver transplantation, Corticosteroids, Nationwide study

## Abstract

**Purpose:**

To provide updated nationwide outcomes of biliary atresia (BA) among patients in Taiwan, focusing on postoperative cholangitis, liver transplantation (LT), corticosteroid use, and renal outcomes.

**Methods:**

Infants with BA who underwent Kasai portoenterostomy were identified from the Taiwan National Health Insurance Research Database (2008–2019). Postoperative outcomes were evaluated, and the association between cholangitis episodes and LT was analyzed using exploratory logistic regression analysis. Corticosteroid exposure was categorized by duration, and acute kidney injury was assessed as a secondary outcome.

**Results:**

This study included 334 infants. Acute cholangitis occurred in 76.3% of cases, with 24.9% experiencing ≥ 5 hospitalizations. A greater number of cholangitis episodes was associated with a higher likelihood of LT (odds ratio: 1.08, 95% confidence interval: 1.00–1.16, *p* = 0.044). Approximately 30.8% of patients developed biliary cirrhosis, and 33.8% received LT. Corticosteroids were prescribed to 96% of patients, and a longer treatment duration was associated with higher complication rates. About 1.8% of patients developed acute kidney injury, with no progression to end-stage renal disease.

**Conclusion:**

Postoperative cholangitis remains an important clinical factor associated with LT in infants with BA. Corticosteroid treatment duration may reflect underlying disease severity rather than therapeutic benefit. Renal complications are uncommon. Optimizing postoperative care—particularly timely cholangitis management—is essential to improve outcomes.

## Introduction

 Biliary atresia (BA) is a progressive obliterative cholangiopathy that leads to biliary cirrhosis. If BA is left untreated, death can occur within the first 2 years of life [1]. BA may develop in isolation or in association with congenital anomalies, particularly involving the cardiac and gastrointestinal systems, in approximately 10–20% of affected infants [2]. The condition remains the leading indication for liver transplantation (LT) in children [3], and its outcomes are strongly dependent on early diagnosis and timely Kasai portoenterostomy. The global incidence of BA ranges from 1 in 5,000 to 1 in 19,000 live births, with higher rates consistently reported in Asian populations compared with Western populations [4–6].

In 2004, Taiwan implemented a nationwide stool color card screening program to facilitate the early identification of infants with pale stools. This program has significantly improved the referral process and enabled earlier surgical intervention. Early nationwide data from the stool color card registry showed a high sensitivity for BA detection and improved rates of timely Kasai portoenterostomy [7, 8]. A population-based study using data from the Taiwan National Health Insurance Research Database (NHIRD) from 1997 to 2011 further confirmed a decreasing trend in age at diagnosis and the rates of Kasai portoenterostomy after the implementation of this screening strategy [9]. Despite these advancements, the incidence of BA in Taiwan remains relatively high, at approximately 1.5–1.6 per 10,000 live births across different study periods. In the context of declining birth rates, optimizing clinical outcomes and understanding associated comorbidities in affected infants have become increasingly important public health priorities.

Despite improvements in early detection, postoperative complications remain the major determinants of long-term outcomes. Acute cholangitis is one of the most common complications of Kasai portoenterostomy and has been consistently associated with disease progression and the need for LT. Earlier long-term cohort studies from Taiwan have revealed that the frequency of cholangitis and the adequacy of bile flow are key determinants of native liver survival after Kasai portoenterostomy [10, 11]. Various postoperative medical strategies, including prophylactic antibiotics, ursodeoxycholic acid, nutritional support, and corticosteroids, are widely used to improve bile drainage [1]. Prophylactic antibiotics have been used to prevent postoperative cholangitis. However, existing evidence remains limited and inconsistent [12]. In particular, corticosteroids enhance bile flow via anti-inflammatory and immunomodulatory effects. However, their clinical benefit remains controversial, and practice patterns vary significantly [4, 13].

In addition to hepatobiliary outcomes, renal complications have been increasingly recognized as important contributors to prognosis. Acute kidney injury (AKI) has been associated with worse outcomes in pediatric surgical populations and those with critical illness [14]. Infants undergoing Kasai portoenterostomy may be particularly vulnerable due to immature renal function and perioperative factors [15]. A single-center retrospective study reported that AKI after Kasai portoenterostomy was associated with longer intensive care unit stay and increased postoperative morbidity [16]. However, nationwide data on renal outcomes in BA remain limited.

Therefore, this study aimed to provide updated nationwide outcomes of BA in Taiwan using recent NHIRD data, with a focus on postoperative cholangitis and its association with LT, patterns of corticosteroid use, and renal outcomes after Kasai portoenterostomy.

## Methods

### Data source and ethical approval

Taiwan’s National Health Insurance program is a government-operated system that covers > 99% of the population. The claims data, including both inpatient and outpatient records, are maintained in the NHIRD for research purposes. The Institutional Review Board of Hualien Tzu Chi Medical Foundation approved this study. The requirement for informed consent was waived due to the use of de-identified secondary data (IRB111-023-B).

### Participants

A retrospective population-based study was conducted using data from the Taiwan NHIRD between January 2008 and December 2019. Infants diagnosed with BA were identified using the International Classification of Diseases, Ninth Revision, Clinical Modification (ICD-9-CM) code 751.61, which is consistent with previous studies [9, 17]. Patients who underwent Kasai portoenterostomy (ICD-9 procedure code 51.37) were included in the analysis. Patients who underwent primary LT (ICD-9 procedure code 50.5) without prior Kasai portoenterostomy were excluded, as this subgroup represents a distinct clinical course and is typically associated with advanced cirrhosis at presentation [18]. The index date was defined as the first recorded diagnosis of BA.

### Outcomes and measured variables

The primary outcomes included postoperative acute cholangitis (ICD-9-CM code 576.1), biliary cirrhosis (ICD-9-CM code 571.6), portal hypertension (ICD-9-CM code 572.3), and LT. Acute cholangitis was defined as hospitalization due to a diagnosis of cholangitis. The secondary outcomes included AKI (ICD-9-CM code 584.x) and chronic kidney disease (ICD-9-CM code 585) after Kasai portoenterostomy. Considering the small number of renal events, these outcomes were analyzed descriptively.

The baseline characteristics included sex, age at diagnosis, age at Kasai portoenterostomy, and major congenital anomalies, including cardiac anomalies, situs inversus, duodenal atresia, and malrotation. Corticosteroid exposure was identified based on prednisolone (Anatomical Therapeutic Chemical classification code H02AB06) and methylprednisolone (Anatomical Therapeutic Chemical classification code H02AB04) prescriptions after the index date. For steroid-related analyses, patients with outcomes that occurred before steroid initiation and those who did not receive corticosteroids during the index hospitalization were excluded. To allow comparison across predefined exposure-duration categories, patients with corticosteroid use extending beyond one year after the index date were excluded from the duration-based analyses. Then, the patients were categorized according to the duration of corticosteroid use (≤ 90, 91–180, and > 180 days) for outcome comparisons.

### Statistical analysis

The annual incidence of BA was calculated as the number of newly diagnosed cases divided by the number of live births per year. Temporal changes in the incidence of BA were described over the study period. Continuous variables were expressed as the mean ± standard deviation, and categorical variables were presented as the frequencies and percentages. The postoperative outcomes across age groups at Kasai portoenterostomy were compared using the chi-square test. Because acute cholangitis was the most common postoperative complication, an exploratory unadjusted logistic regression analysis was performed to evaluate the relationship between the number of cholangitis episodes and subsequent liver transplantation. Adjustment for potential confounders was not performed because several clinically relevant variables reflecting disease severity were unavailable in the administrative database. For the analyses of corticosteroid use, the patients were classified based on duration of exposure, and differences in outcomes across groups were assessed using the chi-square test, with additional trend analysis across ordered categories. All statistical analyses were performed using the SAS software (version 9.3; SAS Institute, Cary, NC, the USA). *p* < 0.05 was considered statistically significant.

## Results

### Demographic characteristics of the patients

**Fig. 1:**
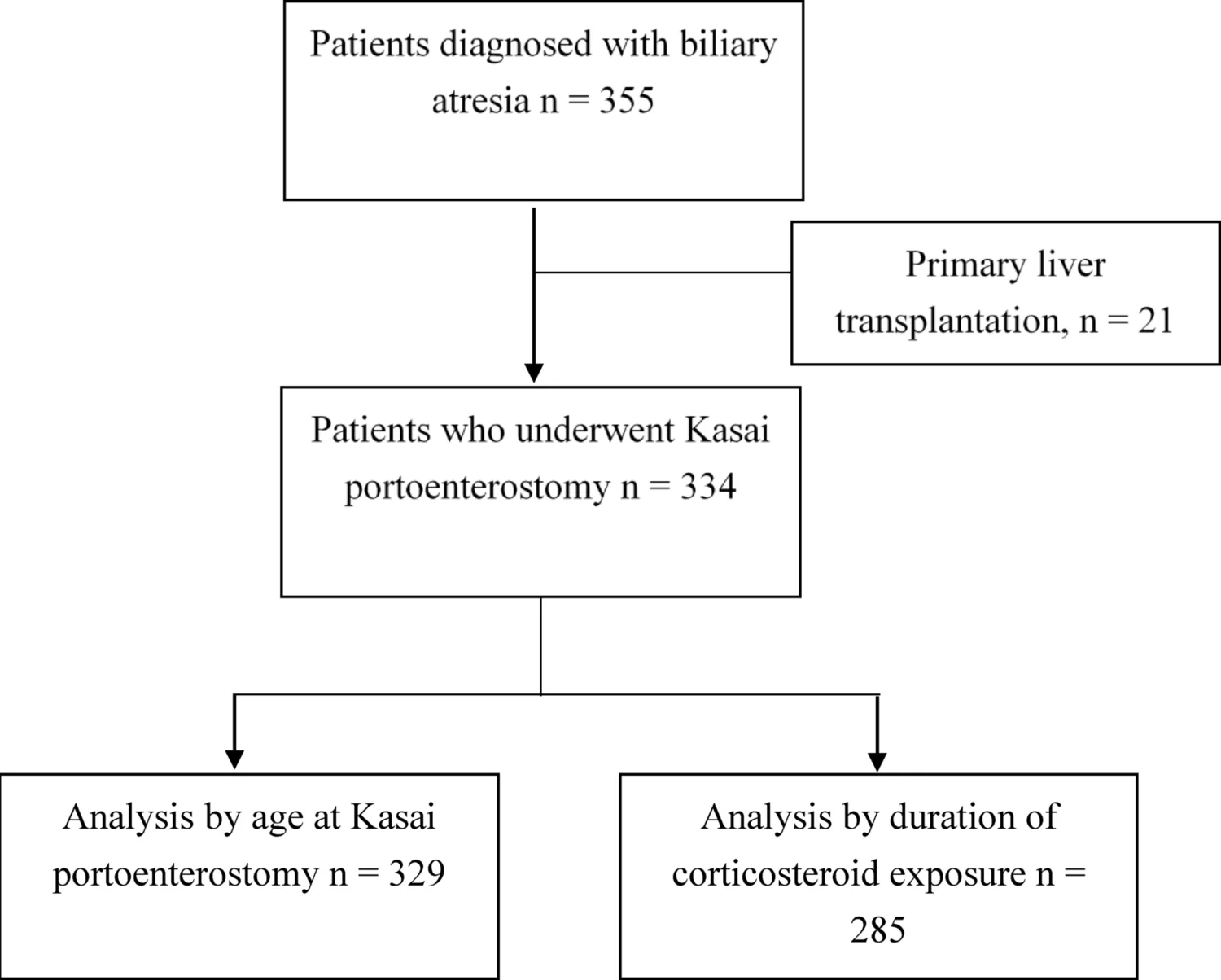
Flow diagram of the study cohort selection Patients with biliary atresia (BA) were identified from the Taiwan National Health Insurance Research Database. After excluding those who received primary liver transplantation without prior Kasai portoenterostomy, 334 patients were included in the analysis. After excluding patients with outcome diagnoses documented prior to the index date (n = 5), 329 patients were included in the analysis based on age at Kasai portoenterostomy. For corticosteroid-related analyses, patients with outcomes documented prior to corticosteroid initiation, those without corticosteroid use during the index hospitalization, and those with corticosteroid use extending beyond 1 year after the index date were excluded, resulting in 285 patients

**Fig. 2:**
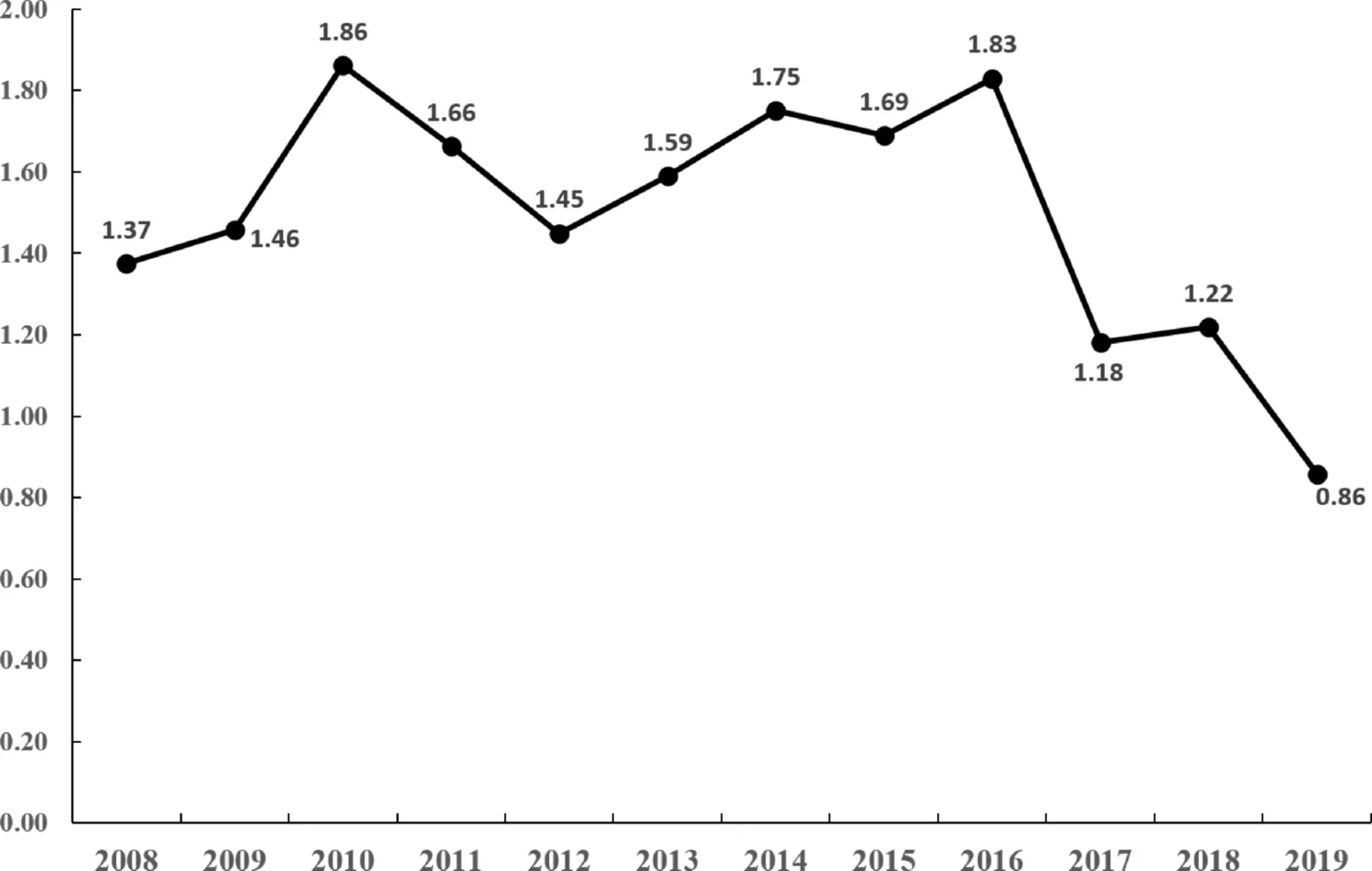
Annual incidence of biliary atresia in Taiwan from 2008 to 2019The annual incidence was calculated as the number of newly diagnosed cases per 10,000 live births based on data from the NHIRD

In total, 355 patients diagnosed with BA were identified during the study period. Among them, 21 (5.9%) received primary LT without prior Kasai portoenterostomy. After excluding patients who received primary LT, 334 infants who underwent Kasai portoenterostomy between 2008 and 2019 were included in the final analysis (Fig. 1). Table 1 presents the baseline characteristics of the study cohort. Female patients accounted for 55.4% of the cohort. The annual incidence of BA ranged from 0.86 to 1.86 per 10,000 live births during the study period (Fig. 2) and remained relatively stable over time, with a slight decline observed in the later years.

**Table 1:** Baseline characteristics and clinical outcomes of patients with biliary atresia who underwent Kasai portoenterostomy

Characteristics of the patients	All patients N = 334 (%)
*Sex*	
Male	149 (44.6%)
Female	185 (55.4%)
*Age at diagnosis*	
0–30 days	96 (28.7%)
31–60 days	152 (45.5%)
61–90 days	78 (23.4%)
>91 days	8 (2.4%)
Age at Kasai portoenterostomy Comorbidity	49.5 ± 49.7
*Cardiac anomaly*	57 (17.1%)
Situs inversus	3 (0.9%)
Duodenal atresia	4 (1.2%)
Malrotation	4 (1.2%)
*Outcomes*	
Acute cholangitis	255 (76.3%)
Biliary cirrhosis	103 (30.8%)
Portal hypertension	67 (20.1%)
Liver transplantation	113 (33.8%)
Perioperative AKI	6 (1.8%)

Data are presented as number (%) or mean ± standard deviation. Congenital anomalies include cardiac anomalies, situs inversus, duodenal atresia, and intestinal malrotation. Acute cholangitis was defined as hospitalization with a diagnosis of cholangitis. AKI was identified using ICD-9-CM code 584.x

The mean age at Kasai portoenterostomy was 49.5 ± 49.7 days (range, 0–613), and the mean age at subsequent LT was 1.39 ± 1.53 years (range, 0.18–7.1). The wide standard deviation reflected a small number of patients who underwent Kasai portoenterostomy at a substantially delayed age, resulting in a skewed age distribution. Sixty-eight (20.4%) patients had congenital anomalies, including congenital heart disease (17.1%), situs inversus (0.9%), duodenal atresia (1.2%), and intestinal malrotation (1.2%). During follow-up, 255 (76.3%) patients experienced at least one episode of acute cholangitis, and 103 (30.8%) developed biliary cirrhosis. Further, 67 (20.1%) presented with portal hypertension, and 113 (33.8%) received LT. Regarding the secondary outcomes of Kasai portoenterostomy, postoperative AKI was rarely observed (*n* = 6, 1.8%). No cases of progression to end-stage renal disease were identified during follow-up.

### Age at Kasai portoenterostomy and outcomes

To evaluate postoperative outcomes, patients with documented outcome diagnoses prior to the index date were excluded, leaving 329 patients for analysis. The distribution of postoperative outcomes did not differ significantly across age groups stratified by the timing of Kasai portoenterostomy, as shown in Table 2. Acute cholangitis occurred in 115 infants who underwent surgery at ≤ 40 days old, in 70 infants at 41–60 days old, and in 70 infants at > 60 days old (*p* = 0.141). The three groups did not significantly differ in terms of the incidence of biliary cirrhosis and portal hypertension and the rates of LT. Overall, no statistically significant differences in postoperative outcomes were observed among the age groups in this cohort. Among patients who developed acute cholangitis, 101 (39.6%) subsequently underwent LT. In exploratory logistic regression analysis, a greater number of cholangitis episodes was associated with a higher likelihood of LT (odds ratio [OR], 1.08; 95% confidence interval [CI] 1.00–1.16, *p* = 0.044).

**Table 2:** Postoperative outcomes according to age at Kasai portoenterostomy (*N* = 329)

Outcomes	Kasai portoenterostomy at ≤40 days old n = 158	Kasai portoenterostomy at 41–60 days old n = 86	Kasai portoenterostomy at >60 days old n = 85	P value
Acute cholangitis	115 (72.8%)	70 (81.4%)	70 (82.3%)	0.1414
Biliary cirrhosis	47 (29.7%)	22 (25.6%)	31 (36.5%)	0.2928
Portal hypertension	26 (16.5%)	15 (17.4%)	24 (28.2%)	0.0731
Liver transplantation	46 (29.1%)	33 (38.4%)	34 (40%)	0.1540

Data are presented as number (%). Patients were stratified by age at Kasai portoenterostomy (≤40, 41–60, and >60 days). Outcomes included acute cholangitis, biliary cirrhosis, portal hypertension, and liver transplantation. Patients with outcome diagnoses documented prior to the index date were excluded from the corresponding analyses. Comparisons were performed using the chi-square test. *p* < 0.05 was considered statistically significant

### Corticosteroid use and outcomes

Considering the high burden of postoperative cholangitis, the role of corticosteroid use in this cohort was further evaluated. Postoperative corticosteroids were prescribed to 96% of patients. Among the 334 patients, 5 patients with outcomes documented prior to corticosteroid initiation were excluded from the steroid-related analyses. For steroid-related analyses, patients who did not receive corticosteroids during the index hospitalization were further excluded, leaving 285 patients for the corticosteroid duration analysis. The mean duration of corticosteroid use was 131 ± 248 days. In the overall cohort, the mean time to discontinuation of corticosteroid use after Kasai portoenterostomy was 2.2 ± 2.6 years. Among these patients, 221 (77.5%) developed acute cholangitis, of whom 55 (24.9%) experienced more than five hospitalizations. Most episodes (85.9%) occurred within the first postoperative year, and 10.5% of the patients had more than five recurrent episodes during this period.

For the subgroup analysis, the patients were categorized into three groups according to the duration of corticosteroid exposure, defined as the timing of the last prescription within 1 year after the index date: ≤90, 91–180, and > 180 days. When categorized according to corticosteroid exposure duration, patients with longer corticosteroid use more frequently experienced acute cholangitis, biliary cirrhosis, and LT (Table 3), with a significant trend across duration groups. However, these findings likely reflected a greater disease severity or persistent cholestasis rather than a direct adverse effect of corticosteroid therapy.

**Table 3:** Postoperative outcomes according to the duration of corticosteroid exposure (*n* = 285)

Outcomes	≤90 days n = 164	91–180 days n = 37	>180 days n = 84	P value
Acute cholangitis	117 (71.3%)	29 (78.4%)	75 (89.3%)	0.0058
Biliary cirrhosis	41 (25%)	18 (48.6%)	28 (33.3%)	0.0150
Portal hypertension	29 (17.7%)	7 (18.9%)	22 (26.2%)	0.2817
Liver transplantation	25 (15.2%)	10 (27%)	65 (77.4%)	<.0001

Data are presented as number (%). Among patients receiving postoperative corticosteroids, those with outcomes documented prior to corticosteroid initiation and those who did not receive corticosteroids during the index hospitalization were excluded. Patients with corticosteroid use extending beyond 1 year after the index date were also excluded from the duration-based analysis. A total of 285 patients were included. Patients were categorized by duration of corticosteroid exposure based on the timing of the last prescription within 1 year after the index date (≤90, 91–180, and >180 days). Outcomes included acute cholangitis, biliary cirrhosis, portal hypertension, and liver transplantation. Comparisons were performed using the chi-square test. *p* < 0.05 was considered statistically significant

## Discussion

In this contemporary cohort, acute cholangitis occurred in 76.3% of patients after Kasai portoenterostomy, and a greater number of cholangitis episodes was associated with a higher likelihood of liver transplantation (OR, 1.08; 95% CI, 1.00–1.16). No statistically significant differences in postoperative outcomes were observed across age groups stratified by timing of Kasai portoenterostomy. BA remains a major indication for pediatric LT, and early diagnosis with timely Kasai portoenterostomy is essential for improving patient outcomes. In Taiwan, the nationwide stool color card screening program has reduced the age at diagnosis and facilitated earlier surgical intervention, as shown in previous registry- and population-based studies [7–9]. According to our findings, this benefit has been sustained, as reflected by a modest increase in the proportion of patients undergoing Kasai portoenterostomy within 60 days.

The apparent decline in the incidence of BA in the most recent years should be interpreted with caution, as it may reflect incomplete case ascertainment related to delayed diagnosis or limitations in data capture within administrative databases. Previous studies have consistently shown that earlier Kasai portoenterostomy is associated with improved native liver survival. However, the reported success rates vary based on patient characteristics and follow-up duration. Age at Kasai portoenterostomy is associated not only with native liver survival but also with the severity of hepatic fibrosis at initial biopsy and the morphology of biliary remnants at surgery, thereby underscoring the importance of early referral and timely intervention [10, 11]. For example, Wang et al. reported improved native liver survival if surgery was performed before 81 days of age. However, a large multicenter study conducted in France revealed 5-, 10-, and 20-year native liver survival rates of 40%, 36%, and 30%, respectively [19, 20]. In current practice, postoperative management focuses not only on surgical timing but also on preventing complications—particularly cholangitis—and optimizing nutritional support [1, 21].

Despite these advancements, postoperative complications remain common and continue to affect long-term outcomes. In our cohort, acute cholangitis was highly prevalent and was associated with subsequent LT. However, this finding should be interpreted as an observational association rather than evidence of an independent or causal relationship, given the lack of adjustment for disease severity and other potential confounders. Most episodes occurred within the first postoperative year, which is consistent with previous reports [22]. Indeed, cholangitis is most frequently observed within the first 2 years of life after Kasai portoenterostomy, and it remains a major driver of progressive hepatic injury [11]. The underlying mechanisms are likely multifactorial, including ascending infection via portoenterostomy, bile stasis within dilated intrahepatic ducts, and ongoing inflammatory injury. Recurrent episodes may accelerate fibrosis and contribute to progressive hepatic injury and biliary cirrhosis, ultimately leading to LT [23]. A subset of patients experienced frequent recurrent episodes, indicating a particularly high complication burden. In long-term survivors, portal hypertension and recurrent cholangitis are common complications and may contribute to adverse long-term outcomes, including mortality and LT [10]. LT is often required within the first 2 years of life in patients with poor bile drainage after Kasai portoenterostomy and may still be required later due to progressive disease and refractory biliary tract infection [10]. These findings are consistent with long-term cohort studies from Taiwan, which have shown that recurrent cholangitis and inadequate bile drainage are strongly associated with worse native liver survival after Kasai portoenterostomy [10, 11].

In contrast to earlier studies, we did not observe statistically significant differences in postoperative complications or LT across age groups stratified by timing of Kasai portoenterostomy. However, these findings should be interpreted cautiously. The study may have been underpowered to detect modest differences between groups, and residual confounding cannot be excluded. In addition, native liver survival could not be directly assessed in the NHIRD, and liver transplantation was used as a surrogate outcome, which may not fully capture the long-term impact of surgical timing. Improvements in perioperative care and closer postoperative surveillance may have reduced differences between early and later surgical groups. In addition, the increasing availability of LT, including living donor transplantation, may allow earlier transplantation in patients with suboptimal response to Kasai, thereby attenuating the observable effect of surgical timing [9]. Previous studies have shown that, in addition to surgical timing, factors such as bile flow and the frequency of cholangitis play important roles in determining long-term outcomes [11]. Taken together, these findings suggest that postoperative management—particularly the prevention and timely management of cholangitis—plays a central role in determining long-term outcomes.

Corticosteroid therapy was nearly universal in our cohort, reflecting current clinical practice after Kasai portoenterostomy. Although corticosteroids are thought to enhance bile flow via anti-inflammatory and immunomodulatory effects, clinical evidence regarding their therapeutic benefit remains inconsistent. The proposed mechanisms include enhanced bile acid transport, suppression of immune-mediated inflammation, and inhibition of proinflammatory gene transcription [4]. Early studies by Davenport et al. reported improved jaundice clearance, particularly in infants undergoing early surgery. However, this did not translate into improved native liver survival or reduced LT rates [24, 25]. Similarly, the multicenter START trial revealed no significant improvement in bile drainage or transplant-free survival. However, it observed an increased incidence of adverse events [13]. Previous meta-analyses have shown potential short-term benefits, with inconsistent long-term outcomes and no clear reduction in the incidence of postoperative cholangitis [4, 26]. More recently, a randomized controlled trial reported improved bile drainage and native liver survival with adjuvant steroid therapy without a significant increase in early adverse events. Nevertheless, these findings differ from those of earlier trials [27]. Emerging evidence further indicates that patients undergoing earlier Kasai portoenterostomy (≤ 70 days) may experience greater therapeutic benefits from steroid therapy. Meanwhile, patients undergoing later surgery may be less responsive due to more advanced hepatic injury [4].

In our cohort, corticosteroid use was not only widespread but also highly variable, with a mean treatment duration of 131 ± 248 days and a mean time to cessation of 2.2 ± 2.6 years after surgery. This variability likely reflects differences in clinical decision-making and underlying disease course. Consistent with this interpretation, patients receiving longer courses of corticosteroids were more likely to develop cholangitis, biliary cirrhosis, and LT during follow-up. However, this observation should be interpreted cautiously, as treatment duration was determined by clinical practice rather than random allocation and is therefore highly susceptible to confounding by indication and time-dependent bias. Patients with more severe or persistent cholestasis are more likely to receive prolonged therapy, which may account for the observed associations. Taken together, these findings indicate that although corticosteroids are still widely used in real-world practice, the treatment duration may reflect disease severity rather than therapeutic benefit. In some centers, corticosteroids are selectively administered based on early postoperative bile drainage, and ongoing research aims to identify the subgroups of patients who may benefit most. Future studies should focus on defining optimal dosing strategies, timing of initiation, and treatment duration and identifying patients most likely to benefit from corticosteroid therapy.

Renal complications were rare in our cohort. Only 1.8% of patients presented with postoperative AKI, and none of the cases involved progression to end-stage renal disease. Although AKI has been increasingly recognized in pediatric liver disease, data specific to BA remain limited. Previous studies have reported higher incidences of AKI after Kasai portoenterostomy, with rates up to 22%, and have identified several perioperative risk factors [16]. In contrast, the incidence of AKI in our study was substantially lower. This discrepancy may reflect differences in study design, patient selection, and case ascertainment. Notably, the ICD-based identification of AKI has a limited sensitivity compared with clinically defined criteria, particularly for mild or transient AKI, which may lead to underestimation in administrative database studies [28, 29]. Therefore, the renal findings should be considered exploratory and interpreted with caution. Given the small number of events, further analysis was not feasible.

Biliary cirrhosis and portal hypertension were observed in a subset of patients during the study period, although these rates are likely underestimated due to the limited follow-up data. In long-term clinical series, both conditions are more frequently identified in later childhood and adolescence, reflecting their progressive nature. Together with recurrent cholangitis, these complications contribute to late-onset liver transplantation, underscoring the importance of continued long-term surveillance beyond the immediate postoperative period [10, 11].

This study has several limitations. First, the use of administrative data might have introduced coding inaccuracies and lacked detailed clinical information, such as laboratory parameters, bile drainage status, and disease severity. In addition, individual follow-up duration was not available for the current analysis, which may have influenced the assessment of long-term outcomes such as liver transplantation and biliary cirrhosis. Second, the observational design precludes causal inference, particularly in the evaluation of corticosteroid use. Third, perioperative variables and surgical details were not available. In addition, native liver survival could not be directly assessed due to database limitations, including the lack of detailed longitudinal follow-up and precise timing of clinical events. Therefore, LT was used as a clinically relevant surrogate outcome, which is consistent with previous NHIRD-based studies in this population [9, 30]. Finally, the low number of AKI events limited further analysis of associated risk factors.

In conclusion, despite sustained benefits from nationwide screening in Taiwan, postoperative cholangitis remains an important clinical factor associated with LT in infants with BA. Corticosteroid use is nearly universal. However, treatment duration may reflect underlying disease severity rather than therapeutic benefit. Renal complications are rarely observed. These findings emphasize the importance of optimizing postoperative care—particularly the prevention and timely management of cholangitis—while further validating the role of corticosteroid therapy in improving long-term outcomes. From a surgical perspective, early diagnosis and aggressive management of postoperative cholangitis may be a key modifiable factor for improving transplant-free survival.

## Data Availability

All data generated or analyzed during this study are included in this published article.
